# Improving physio-mechanical and biological properties of 3D-printed PLA scaffolds via in-situ argon cold plasma treatment

**DOI:** 10.1038/s41598-023-41226-x

**Published:** 2023-08-29

**Authors:** Masoud Zarei, Sayed Shahab Sayedain, Amirhossein Askarinya, Mobina Sabbaghi, Reza Alizadeh

**Affiliations:** Department of Materials Science and Engineering, Sharif University of Technology, Azadi Ave., Tehran, 11155-9466 Iran

**Keywords:** Tissues, Mechanical properties, Biomedical engineering

## Abstract

As a bone tissue engineering material, polylactic acid (PLA) has received significant attention and interest due to its ease of processing and biocompatibility. However, its insufficient mechanical properties and poor wettability are two major drawbacks that limit its extensive use. For this purpose, the present study uses in-situ cold argon plasma treatment coupled with a fused deposition modeling printer to enhance the physio-mechanical and biological behavior of 3D-printed PLA scaffolds. Following plasma treatment, field emission scanning electron microscopy images indicated that the surface of the modified scaffold became rough, and the interlayer bonding was enhanced. This resulted in an improvement in the tensile properties of samples printed in the X, Y, and Z directions, with the enhancement being more significant in the Z direction. Additionally, the root mean square value of PLA scaffolds increased (up to 70-fold) after plasma treatment. X-ray photoelectron spectroscopy analysis demonstrated that the plasma technique increased the intensity of oxygen-containing bonds, thereby reducing the water contact angle from 92.5° to 42.1°. The in-vitro degradation study also demonstrated that argon plasma treatment resulted in a 77% increase in PLA scaffold degradation rate. Furthermore, the modified scaffold improved the viability, attachment, and proliferation of human adipose-derived stem cells. These findings suggest that in-situ argon plasma treatment may be a facile and effective method for improving the properties of 3D-printed parts for bone tissue engineering and other applications.

## Introduction

Tissue engineering methods based on repairing bone defects have been widely used to treat common and uncomplicated fractures^1^. Today, metallic scaffolds are extensively used for bone tissue regeneration applications, while titanium and titanium alloys are of particular interest for this purpose. Such metallic implants suffer from some inherent weaknesses, such as aseptic inflammation, corrosion, and inability to degrade, where the last one means that the scaffold would remain in the body after implantation and require removal through a secondary surgery^2^. In addition, most metals exhibit higher elastic modulus than human bones, causing the stress-shielding effect^3^. Accordingly, there has been a substantial increase in the use of biodegradable polymers in tissue engineering^4^. Polymeric scaffolds, with high surface-to-volume ratios, high porosity with very small pores, biodegradability, and non-toxicity properties, are drawing considerable attention^5^.

Today, conventional and additive manufacturing (AM) techniques are commonly used to fabricate 3D scaffolds for tissue engineering^6–9^. Conventional techniques, however, present some limitations, such as the high cytotoxicity of organic solvents as well as the difficulty in controlling scaffold microstructure and accuracy^10–12^. On the other hand, additive manufacturing techniques do not require cytotoxic organic solvents and are capable of precisely controlling pore size and neat geometry^13, 14^. Among different AM methods, stereolithography (SLA), fused deposition molding (FDM) or extrusion based printing processes, selective laser sintering (SLS), and 3D bioprinting have been successfully used to make 3D scaffolds. Nowadays, FDM, or extrusion based printing technique, is the most commonly used AM technique in different fields, such as electronics, architecture, and tissue engineering^15–17^. In this process, a polymer-based material is melted by going through the printer nozzle and printed on a moving platform, where it solidifies.

Several thermoplastic polymers are commonly used in the FDM process for bone tissue engineering applications, including polycaprolactone (PCL), poly(glycolic acid) (PGA), polylactic acid (PLA), poly lactic-co-glycolic acid (PLGA) copolymers^18–20^. Among these bio-polymers, PLA is a popular biodegradable and renewable polymer that has gained significant attention in recent years due to its desirable properties, such as superior biocompatibility, processability, and low toxicity^21^. Biomedical applications of this polymer include drug delivery, tissue engineering, and wound healing^22^. Like all engineering materials, PLA also has its own limitations, among them it can be referred to weak mechanical properties, inadequate surface wettability, and low degradation rate, which all restrict its extensive use in biomedical applications^23–25^.

Various approaches have been undertaken in order to overcome these limitations, including addition of metallic^26^, ceramic^27^, or natural polymer^28^ fillers to PLA, application of surface modification techniques, such as biofunctionalization^29, 30^, and coating^31^. Even though these strategies are intended to improve implant’s mechanical properties and biocompatibility through biomolecular and chemical functionalization, they are usually too expensive, time-consuming, and complicated to be used in orthopedic surgeries (multiple procedures)^32, 33^. Therefore, a simplified and more efficient approach would be of particular interest to enhance 3D-printed scaffolds' performance in bone tissue applications.

In contrast to the above-mentioned processes, plasma treatment creates nano-roughness on scaffold surfaces, alters wettability, and enriches surfaces with functional groups, including nitrogen and oxygen atoms, at a lower fabrication cost and level of complexity^34–36^. For example, it has been reported^37^ that surface modification of 3D-printed PLGA/Collagen scaffolds by plasma treatment (with helium gas) resulted in a decrease in water contact angle from 70° to 42°, which decreased the protein release from the scaffold into the phosphate-buffered saline (PBS) solution. In another study, it was shown^38^ that the oxygen and nitrogen plasma treatments could render hydrophilicity of the 3D-printed PCL/hydroxyapatite (HA) /magnesium oxide (MgO) scaffolds and thus, improved the adhesion, proliferation, and differentiation of preosteoblast cells^38^. Also, recently low-pressure plasma with air and oxygen was applied on 3D-printed PLA-TiAl4V composite scaffolds and it was found that surface roughness, wettability, and the intensity of oxygen-containing bonds were all improved as the result of used plasma treatment, and as the result, the biological performance of Wharton’s jelly mesenchymal stem cells (WJ-MSCs) was greatly enhanced^39^.

It should be noted, however, that despite the promising results regarding the biological response of scaffolds, none of the referred studies examined the effect of plasma treatment on mechanical, integrity, and degradability properties. Furthermore, most of the studies in this area, pertaining to 3D-printing and bone tissue engineering, have performed plasma treatment as a secondary process on the surface of fabricated scaffolds. However, to the best of our knowledge, in-situ plasma treatment during the printing process has not been performed yet. Thus, further investigations are required to establish a more accurate correlation between in-situ plasma treatment during the printing process and the corresponding effects on the mechanical, physio-chemical, as well as biological behavior.

Accordingly, this investigation aims to evaluate the effect of in-situ argon plasma treatment on different properties of PLA scaffolds 3D-printed by the FDM. Following printing, modified scaffolds were characterized in terms of mechanical properties in different printing orientations, to study the effect of plasma treatment on the anisotropy of mechanical properties. In addition, the physicochemical, in-vitro degradation and adhesion properties were evaluated along with studying the proliferation behavior of human adipose-derived stem cells (hADSCs).

## Materials and methods

### Filament fabrication

Initially, the received PLA (4032D, NatureWork LLC, USA) pellets were heated in an oven at 65 °C for 12 h in order to remove any moisture. Following dehydration, the PLA pellets were fed into a single-screw extruder. This in-house extruder featured two heat zones, both of which were set to 185 °C during the experiments. Additionally, the screw speed of the extruder was maintained at 30 rpm. These extruding parameters were empirically obtained to produce uniform PLA filament with an average diameter of 1.75 ± 0.25 mm. When the extruding parameters are set to smaller values than the optimized values, several undesirable outcomes can occur. Firstly, the filament may have a reduced diameter or inconsistent thickness along its length. This can lead to variations in mechanical properties and compromise the overall quality of the filament. Additionally, poor adhesion between layers of the printed parts may occur, resulting in a weaker final printed product. On the other hand, when the extruding parameters are set to values that are greater than the optimized values, different problems might arise. The filament diameter may increase, leading to a thicker filament than intended. This can create difficulties in feeding the filament into the 3D printer and may cause blockages during printing, affecting the overall printing performance. The excessive heat or pressure applied during extrusion may cause filament deformation or warping, resulting in dimensional inaccuracies or a poor surface finish in the final printed object.

### 3D-printing of PLA scaffolds

An FDM-based 3D printer (Dayan K12S, Iran) with a nozzle diameter of 400 µm was used to print PLA parts by layer-upon-layer deposition of the fused filament. The overall dimensions of the printed 3D porous scaffolds are 10 mm × 10 mm × 10 mm, while the scaffold's pore size, strut width, and porosity percentage are 500 µm, 600 µm, and 45%, respectively. In Table 1, the used parameters of the FDM printing technique are summarized.

**Table 1:** Process parameters used for printing by the FDM method.

Parameter	Value
Bed temperature	55 °C
Nozzle temperature	200 °C
Printing speed	10 mm/s
Layer height	250 µm
Lay down pattern	− 45°/+ 45°

### In-situ argon atmospheric plasma treatment procedure

While printing samples, they were exposed to an atmospheric cold plasma system developed by our group (Alizadeh research group, Sharif University of Technology). The used torch is characterized by a quartz tube with an inner diameter of 2.5 mm and a wall thickness of 1 mm. The torch employs two electrodes: one in the form of a copper foil surrounding the outer wall of the quartz tube, and the other, a 0.5 mm diameter cupper wire positioned inside the tube. These electrodes are separated by the dielectric quartz tube wall.

A high-voltage and high-frequency power supply converts the input AC electrical current from 220 V at an initial frequency of 50 Hz to 20 kHz, providing a voltage range between 0 to 20 kV. By connecting this power supply to the installed electrodes and introducing the desired gas (argon in this case) into the quartz tube, a dielectric barrier discharge (DBD) is generated between the two electrodes, resulting in plasma formation, as the gas flows between them. The plasma gas emerges from a 0.7 mm diameter nozzle located at the end of the quartz tube, thereby creating a column of cold plasma. The length of this plasma column is contingent upon factors such as the gas flow rate, gas type, and operating voltage. In this research, an operating voltage of approximately 15 kV was used, which, as indicated by the IV characteristic of the plasma torch, yields a current of approximately 3.4 mA. The plasma was supplied by purified compressed argon gas pumped at a rate of 1.5 L/min. The gas flow rate was adjusted to achieve a plasma column length ranging from approximately 12 to 15 mm, considering the geometrical constraints imposed by the 3D printer nozzle at a distance of approximately 10 mm from the cold plasma torch nozzle. The argon plasma torch continuously emitted over the samples at a distance of 10 mm during the printing process.

**Figure 1:**
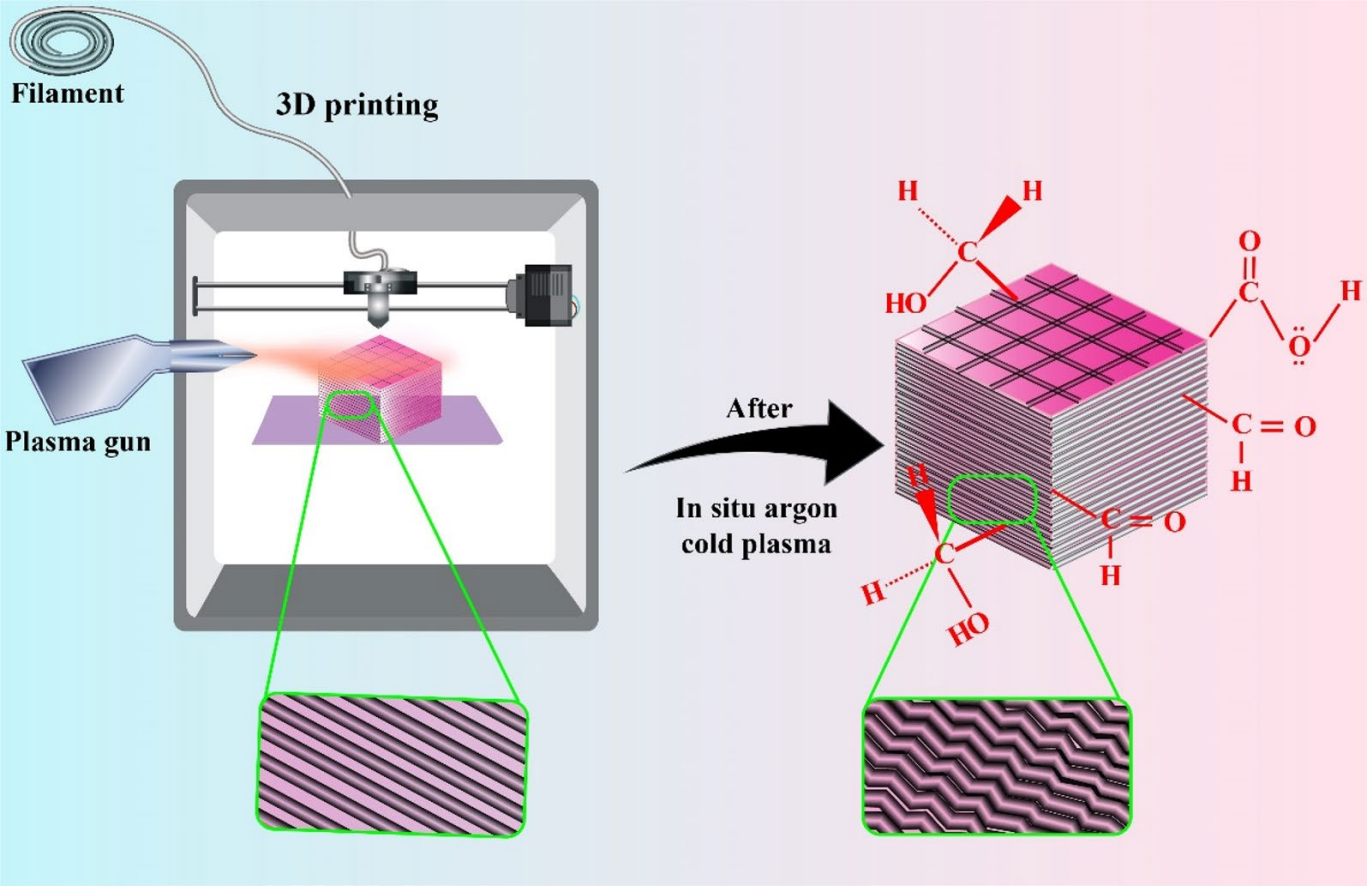
Schematic representation of 3D-printing of PLA treated with in-situ argon cold plasma.

**Figure 2:**
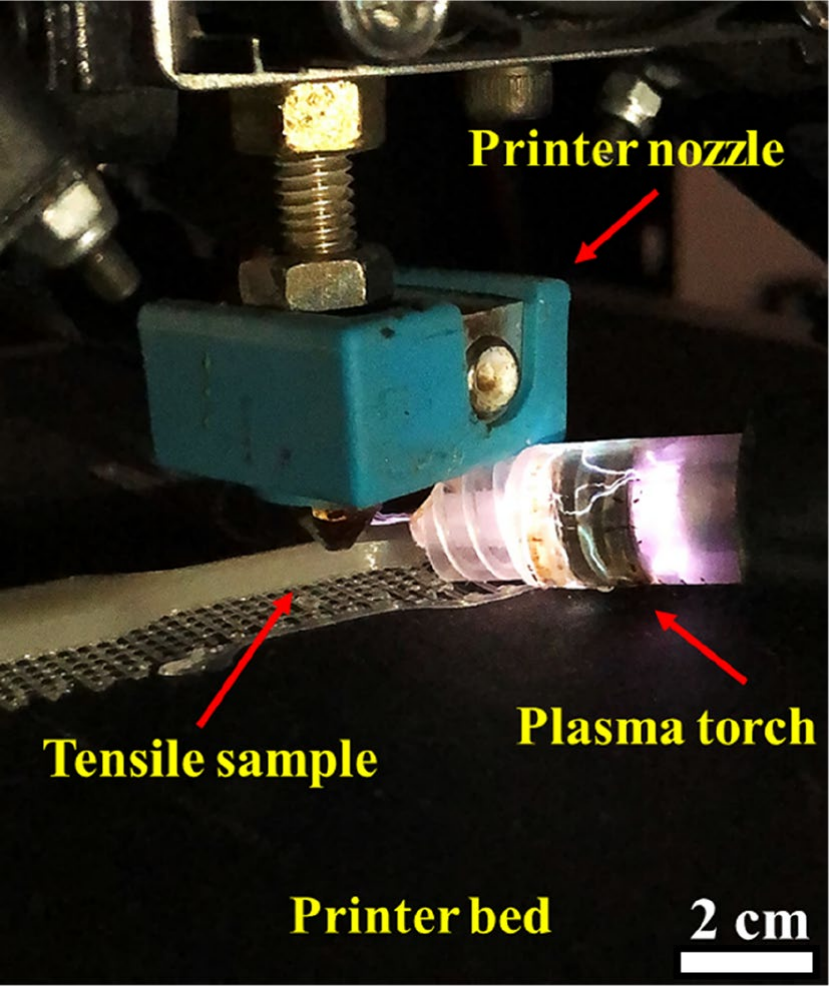
Photograph showing in-situ argon cold plasma treatment during the 3D-printing process.

After plasma treatment, all samples were immediately characterized for further analysis. Some of the samples were left untreated to examine plasma treatment's effect on different properties. A schematic of the procedure is shown in Fig. 1, while a photograph of the in-situ cold argon is presented in Fig. 2.

### Characterization of scaffolds

In order to characterize the microstructural features of the samples, a field emission scanning electron microscope (FESEM, Mira3, Czech Republic) operated at a voltage of 15 kV was utilized. To ensure surface conductivity and prevent buildup of electrical charge during FESEM imaging, samples were initially coated with a thin layer of gold, using sputter coating.

To evaluate the hydrophilicity of untreated and treated samples, contact angle measurements (OCA 15 plus, DataPhysics) were conducted three times on each sample. For this analysis, a drop of deionized water was poured onto the surface, and the contact angle was calculated using the tangent method. In addition, Di-iodomethane (CH2I2, Merck-98%) was employed for the surface free energy (SFE) measurement, using the Owens–Wendt method^40^. According to Eq. (1), ϴ represents the contact angle formed between the surface and the liquid, whereas $\gamma_{s}$,$\gamma_{s}^{d}$ and $\gamma_{s}^{p}$ are the total SFE and its dispersive and polar components, respectively, with the relationship $\gamma_{s} = \gamma_{s}^{p} + \gamma_{s}^{d}$. Table 2 provides the surface tensions associated with the polar and dispersive components.

1 $$\left( {\gamma_{s}^{d} \times \gamma_{l}^{d} } \right)^{0.5} + \left( {\gamma_{s}^{p} \times \gamma_{l}^{p} } \right)^{0.5} = \frac{1}{2}\gamma_{s} \left( {1 + \cos \theta } \right)$$

**Table 2:** Surface tension for polar and dispersive liquids.

Liquids	$\gamma_{l}^{p}$(mN/m)	$\gamma_{l}^{d}$(mN/m)	$\gamma_{l}$(mN/m)
De-ionized water	51.00	21.80	72.80
Di-iodomethane (CH_2_I_2_)	0.01	50.80	50.81

In order to assess the roughness and topography of modified and untreated 3D-printed scaffolds, the atomic force microscopy (AFM, TS-150, NT-MDT, Russia) was performed. The analysis was performed using tapping mode at room temperature on a microscope with a scanning rate of 0.5 Hz for all scanning sizes. A golden silicon probe (NSG 20 series; k = 55 N/m) with a tip curvature of 10 nm was used. The operating point, which determines the oscillation amplitude in tapping mode, was set to 0.172 V. Also, the root mean square (RMS) roughness (Rq) value was computed with the help of NOVA software (version 1.0.26.1443). FTIR spectroscopy (Thermo, US) was employed to investigate the chemical functional groups of PLA scaffolds and their alterations resulting from plasma treatment. The FTIR spectra were obtained using a Perkin Elmer spectrometer within the range of 4000 cm^−1^ to 400 cm^−1^, utilizing attenuated total reflectance (ATR) technique. X-ray photoelectron spectroscopy (XPS, BESTEC, EA 10) was also employed to analyze the surface of untreated and plasma-treated samples. The binding energies of all peaks were calibrated with reference to the C1s core level spectrum position of C\C and C\H (hydrocarbons) species at 284.9 eV. Data acquisition and processing were conducted using the XPSPEAK 4.1 software, employing a Shirley type background subtraction, and peak decomposition utilizing a mixed Gaussian–Lorentzian shape with equal full-width-at-half-maximum.

**Figure 3:**
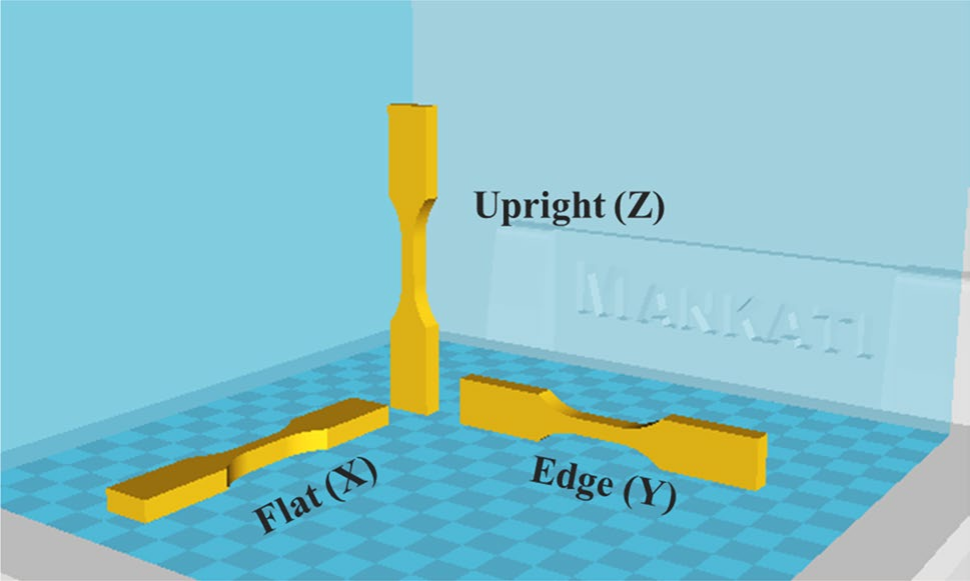
Tensile specimens in different printing orientations.

To evaluate the effect of plasma treatment on mechanical properties, and also anisotropy of mechanical behavior, tensile specimens were printed in the flat (X), edge (Y), and upright (Z) directions according to type V of ASTM D-638 standard (Fig. 3). During printing, argon plasma treatment was applied to some samples in-situ. Tensile specimens could be divided into two groups: untreated and plasma-treated. The untreated samples were referred to as X-NP, Y-NP, and Z-NP, while the plasma-treated ones were referred to as X-PP, Y-PP, and Z-PP. The tensile force was applied using a specific universal testing machine (STM-20, Santam Co., Iran) at a constant crosshead speed of 5 mm/min. The load cell used for force measurement had a capacity of 1000 kg, indicating its ability to accurately measure forces up to 1000 kg during the tensile test. To determine the reproducibility of the data, tensile tests were repeated at least three times, and the mean values were reported.

### In-vitro biodegradation evaluation

Untreated and plasma-treated scaffolds with an initial dry weight of *W*_i_ were immersed in centrifugal tubes filled with 30 mL of PBS solution (pH 7.4). The tubes were placed in an incubator at 37 °C for up to 8 weeks. The samples were taken out of the tubes at predetermined intervals and weighed after being dried in an oven for 10 h at 60 °C to obtain the weight of scaffolds after degradation, *W*_d_. Using a pH meter (Starter ST2200, China), the pH of the degradation solution was recorded. The following equation was used to calculate sample weight loss:

2 $$Weight\;{\text{loss}}(\% ) = \frac{{(W_{i} - W_{d} )}}{{W_{i} }} \times 100$$

The values were expressed as mean ± SD (n = 3). After 8 weeks, 3D-printed scaffolds were studied by FESEM analysis.

### Cell behavior

In this study, human adipose-derived stem cells (hADSCs) purchased from the Pasteur Institute of Iran under the ethics number IBRCC11347 were employed. Following six passages, each well of the 96-well plate was seeded with 1 × 10^4^ cells. To explore the cell behavior on the scaffolds, the supernatant (liquid medium containing unattached or non-viable cells) was diligently removed from each well after 24 h. This meticulous step ensured that the assessment of cell behavior on the scaffold remained unaffected by extraneous factors. The scaffolds were then positioned in close proximity to the cells to foster cell attachment and interaction with the scaffold material. This strategic measure aimed to simulate the in vivo environment, thus enabling the investigation of cell behavior in a context closely resembling the physiological setting. The plates were incubated at 37 °C with 5% CO_2_ in an incubator (Memmert) for two and five days. Throughout this incubation period, the cells were maintained in a conducive physiological environment, promoting their growth and proliferation and allowing them to interact with the scaffold while exhibiting their natural behavior. At the conclusion of the incubation period, the culture medium in each well was discarded, and a culture medium containing 10% MTT (3-(4, 5-Dimethylthiazol-2-yl)-2, 5-diphenyltetrazolium bromide) solution was introduced. The primary purpose of this step was to evaluate cell viability and metabolic activity, and for this purpose, MTT served as a commonly used reagent. The cell plate was then returned to the incubator at 37 °C with 5% CO_2_ for 3–5 h. During this interval, MTT underwent conversion into purple formazan crystals, facilitated by the metabolically active cells. The duration of incubation was meticulously optimized to ensure proper conversion of MTT. Following the formation of purple crystals in the wells, the medium was gradually withdrawn, and 50–70 µl of dimethyl sulfoxide (DMSO) was introduced to each well. DMSO played a crucial role in dissolving the purple crystals, thus releasing the color for subsequent measurements. To ensure the complete dissolution of the purple crystals in DMSO, the cell plate was placed on a shaker (Heidolph) and subjected to shaking for 20 min. This methodical shaking step enabled thorough mixing and homogenization of the solution. Ultimately, the percentage of cell viability was determined by measuring the optical absorption of the solution at a wavelength of 570 nm. The intensity of the purple formazan dye, which directly correlated with the metabolic activity and viability of the cells, was then used to calculate the percentage of viable cells.

To prepare specimens for FESEM analysis, cells were pretreated with glutaraldehyde (2.5 wt %) for an hour, and then rinsed in the PBS solution for an hour. The samples were dehydrated by immersing them in 37 °C for 5 min at each of the following ethanol concentrations (30, 50, 70, 80, 90, 95, and 99%). The samples were then subjected to FESEM analysis after being coated with gold.

This study utilized 4′, 6-diamidino-2-phenylindole (DAPI) staining to study and analyze the cell cycle and to identify the nuclei of the cells. To investigate cell death and determine whether it is of the apoptotic type or not, DAPI staining (Sigma-Aldrich Company, Germany) and fluorescent microscopy (Leica, TCS SP-8, Germany) were employed. The staining indicates that normal cells are blue in color. In spite of this, chromatin condensation and fragmentation of the nucleus render the nuclei of apoptotic cells irregularly visible as bright blue dots^41^. 1 mg of DAPI was dissolved in 1 ml of distilled water to prepare the DAPI solution. Cell death was assessed by culturing hADSCs on the scaffold for an hour before removing the surface of the cells. Following washing with PBS, they were fixed with 4% paraformaldehyde and stained with DAPI at a density of 300 nM.

### Statistical analysis

In this study, all graphs and statistical analyses were carried out using Graphpad Prism 9. T-test analysis was used to calculate P-values and results with *P* < 0.05 were considered statistically significant. Each test was performed in triplicates, and outputs were reported as mean ± standard deviation (SD).

## Results and discussion

### Surface characterization

**Figure 4:**
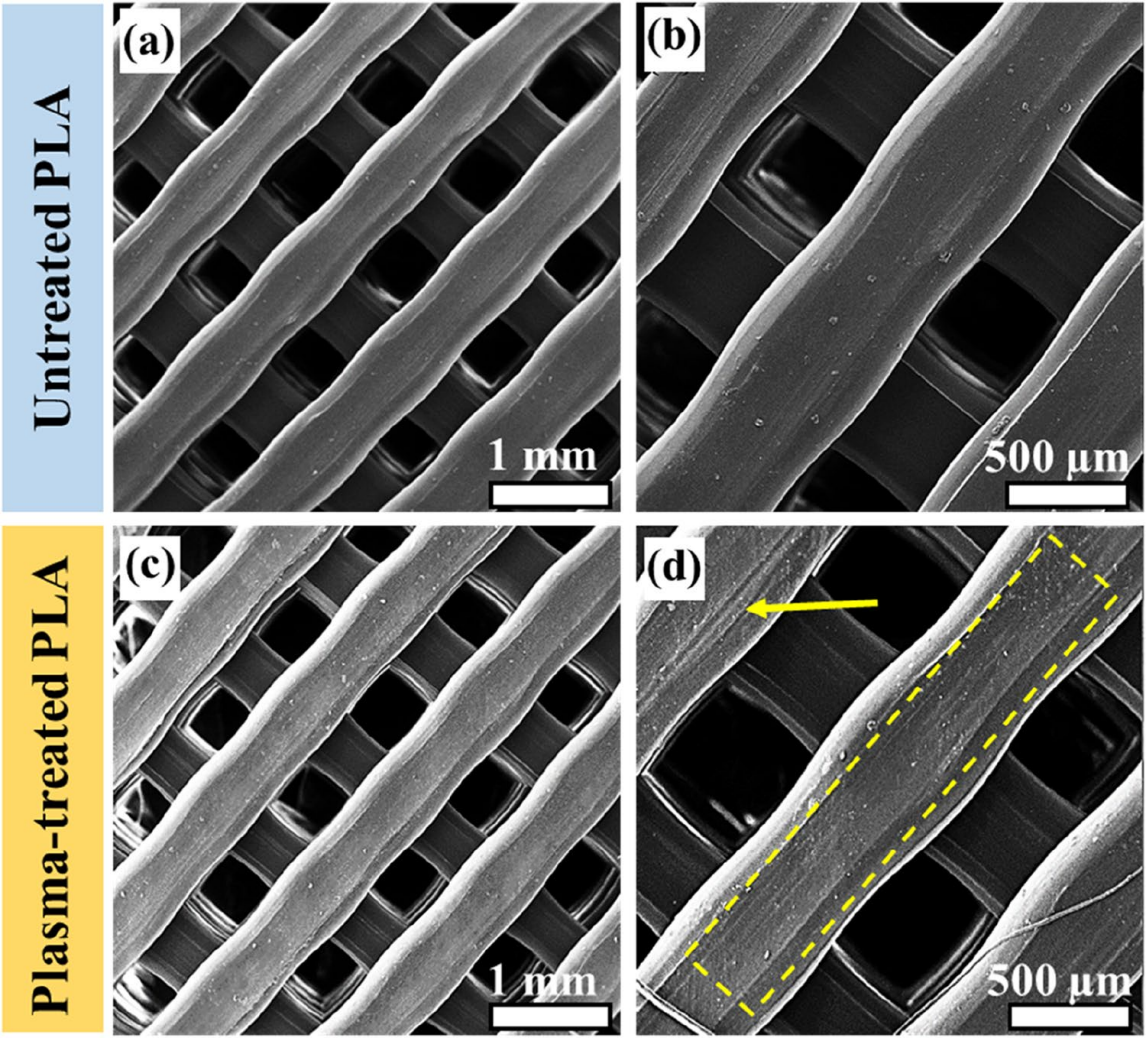
Low and high-magnification FESEM images of untreated (**a**, **b**), and plasma-treated (**c**, **d**) PLA scaffolds.

Scaffolds must have a suitable surface roughness with an open interconnected structure to facilitate the infiltration and growth of cells, as well as transportation of nutrients^42^. Figure 4 demonstrates the FESEM surface topography of untreated and plasma-treated PLA surfaces. As shown in this figure, the 3D-printed scaffolds generally have pore sizes and strut widths of 500 µm, which are within the nominal limits set by the Mankati software, indicating acceptable printing quality. As can be observed in Fig. 4a and b, the untreated PLA scaffold has a smooth surface. The surface morphology of the plasma-treated scaffolds is depicted in Fig. 4c and d, illustrating an increasing amount of nano-patterned roughness on the modified scaffold, as indicated by the yellow dashed line and arrow. The surface etching induced by argon plasma on scaffolds is one of the most pronounced effects of plasma treatment, capable of roughening the surface and producing nano-scale etching spot features^39^. Prolonged plasma treatment may result in heat accumulation in the sample due to collisions between charged particles and surfaces. Thus, the in-situ plasma treatment will increase the scaffold surface temperature to some extent. As a result of this accumulated temperature, the polymer will undergo partial melting and re-forming during plasma treatment, which will allow the surface characteristics to be converted from micro-scale to nano-scale^43^. It has been demonstrated that this nano-scale surface topography induces beneficial cell behavior^44^. Therefore, this in-situ plasma treatment can result in nanoscale structures on 3D-printed PLA scaffolds.

**Figure 5:**
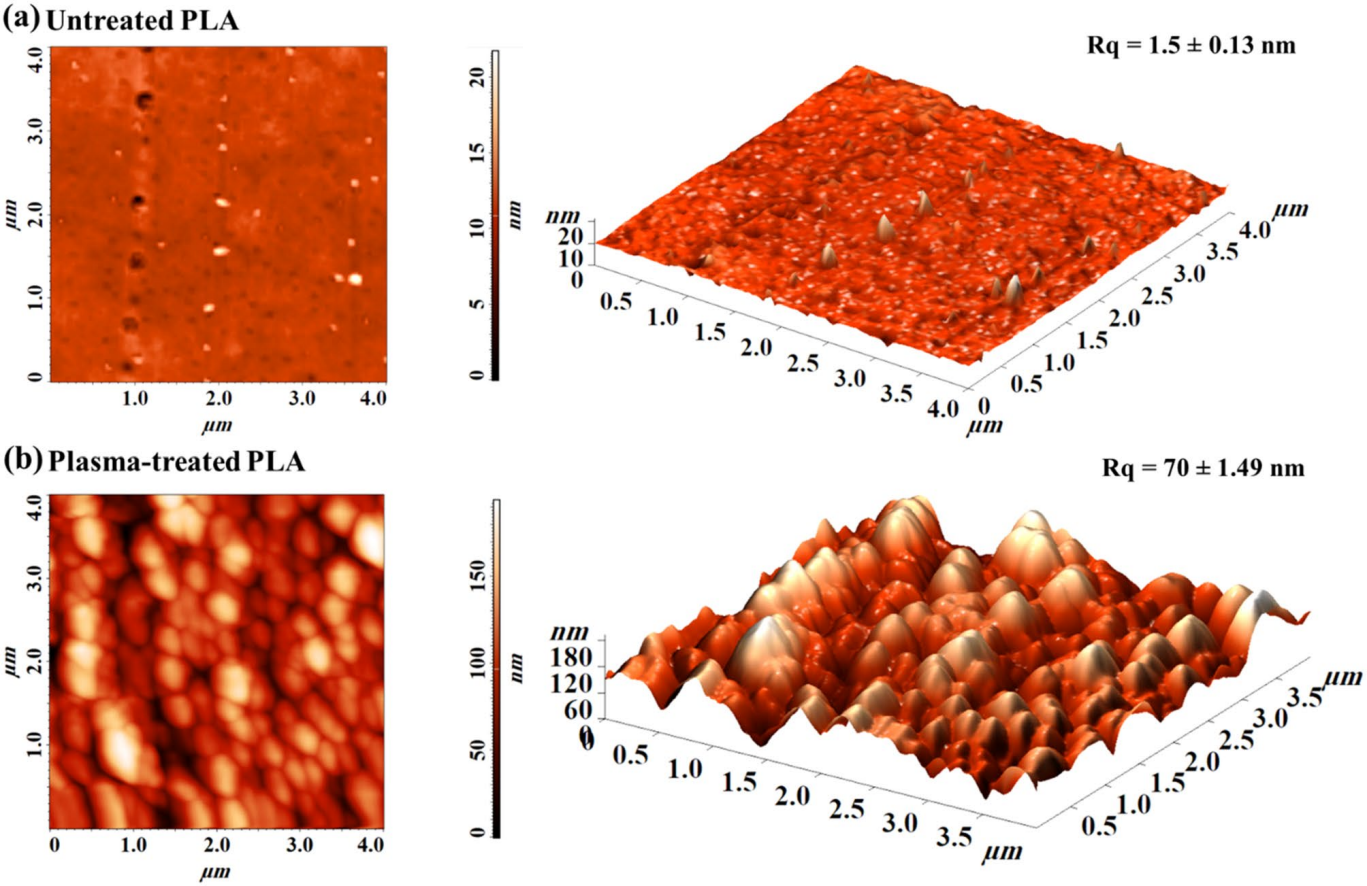
AFM images (from left to right: 2D and 3D) of 3D-printed untreated (**a**) and argon plasma-treated (**b**) PLA scaffolds.

To quantitatively evaluate the created roughness, AFM analysis was employed, where the 2D and 3D plots of plasma-treated and untreated PLA samples are shown in Fig. 5, obtained from a scan window of 4.0 µm × 4.0 µm. In accordance with FESEM results, Fig. 5a shows that untreated PLA exhibits a relatively smooth surface with a roughness (Rq) of 1.5 nm. On the other hand, the plasma-treated scaffold shows uniformly distributed grooves with repetitive peak-valley structures. The Rq value increases significantly after plasma treatment to about 70 nm, as depicted in Fig. 5b.

**Figure 6:**
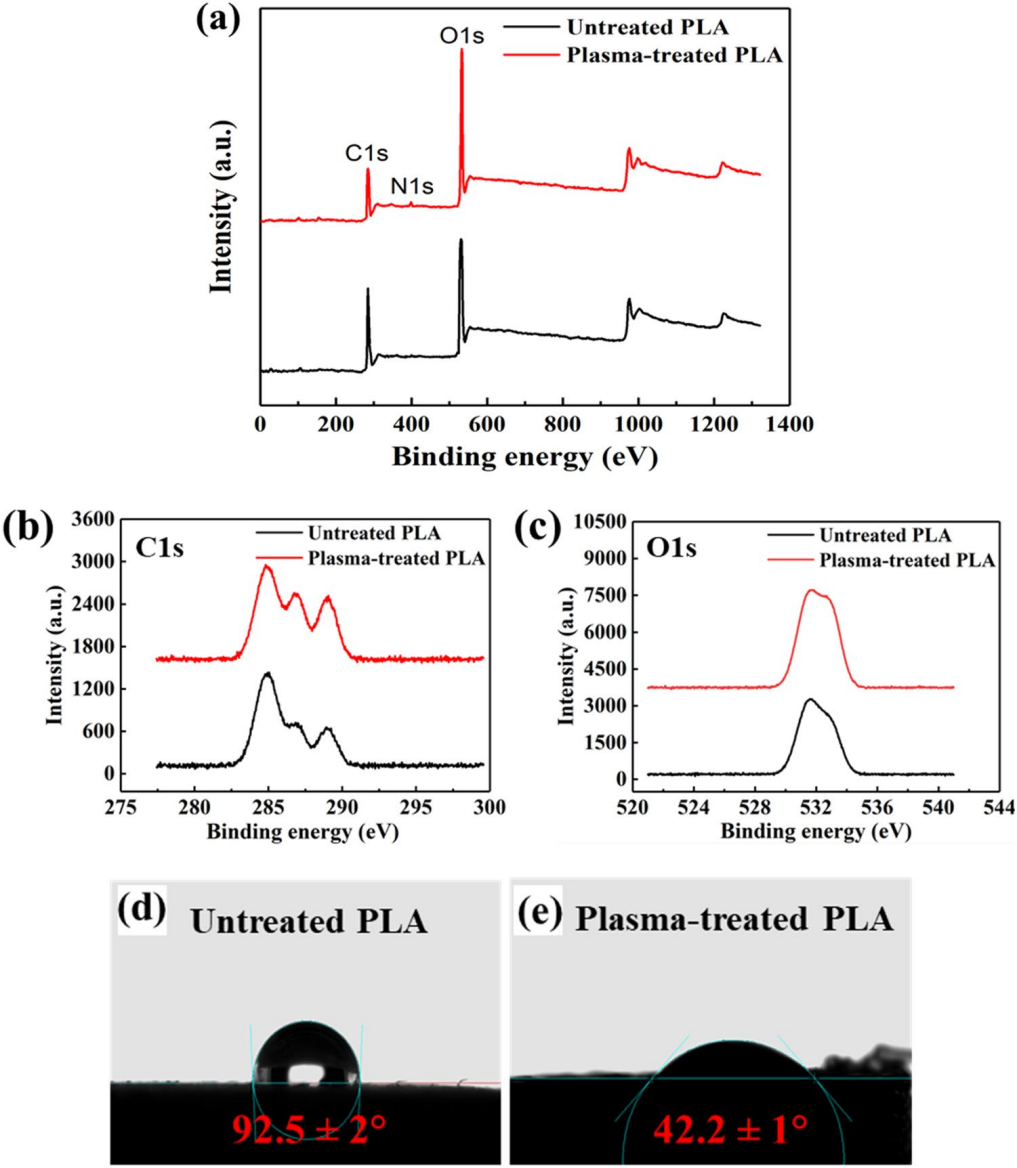
XPS survey scans (**a**), high-resolution XPS spectra of the C1s (**b**), and O1s (**c**) peaks of untreated and plasma-treated PLA scaffolds. The water contact angle of untreated (**d**), and plasma-treated (**e**) PLA scaffolds.

High-resolution XPS analysis was used to compare the untreated and in-situ plasma-treated PLA scaffolds from the chemical state point of view, and the obtained results are shown in Fig. 6 and Table 3. Figure 6a displays survey scans of both the untreated and plasma-treated PLA scaffolds, confirming the presence of carbon and oxygen elements. In addition, after plasma treatment, a small peak at binding energy of 400 eV is appeared. It is noteworthy that although plasma treatment was conducted in the normal atmosphere where nitrogen is more abundant than oxygen, the oxygen-containing functional groups exhibited a more significant reactivity with the PLA sample. This observation can be attributed to the higher electronegativity of oxygen compared to nitrogen^45^. Electronegativity refers to an element's ability to attract electrons in a chemical bond, and oxygen's higher electronegativity indicates a stronger affinity for electrons^46^. As a result of this stronger electron-attracting capability, oxygen is more likely to form new bonds compared to nitrogen during the plasma treatment. In Fig. 6b, deconvoluted C1s spectra of untreated scaffold showed three peaks at 284.9, 286.9, and 289.0 eV, which are associated with the C–H and/or C–C motif, C–O and C–N moiety, as well as C=O bonds, respectively^47, 48^. Similarly, the C1s spectra of the plasma-treated sample presented in Fig. 6b exhibited three peaks relevant to different types of carbon bonds at the same binding energy, but with higher intensity, of the peaks attributed to the C–O (C–N) and C=O. The increase of single and double carbon–oxygen bonds upon plasma treatment substantiated the creation of new chemical bonds on the surface of sample. Such bonds are probably stemmed from the hydroxyl, peroxyl, amines, amides and other polar groups that are conducive to the increase of hydrophilicity as a result of plasma treatment^49^. In Fig. 6c, the O1s spectra of both samples are shown. As evident, two peaks at 531.5 and 533.0 eV can be spotted, which are attributed to the C=O and C–O bonds, respectively^49^. As is obvious, the intensity of both peaks increased for the plasma-treated sample as compared with the untreated one, which is in line with the results of increased oxygen-containing bonds obtained by the C1s spectra. These findings validate the contact angle measurements of the samples. As depicted in Fig. 6d, the untreated PLA scaffold exhibits a contact angle of 92.5°, indicating a hydrophobic surface. However, after the argon plasma treatment, the contact angle decreases significantly to 42.2°, signifying a hydrophilic surface (Fig. 6e).

**Table 3:** Atomic composition and relative composition of the C1s and O1s components for the untreated and plasma-treated 3D-printed PLA samples.

Sample	C1s (%)	O1s (%)	N1s (%)	C1s components (%)			O1s components (%)	
				C–C/C–H	C–O	C=O	O=C	O–C
Untreated PLA	70.5	29.5	0.0	55.0	23.4	21.6	37.5	62.5
Plasma-treated PLA	58.7	40.5	0.8	37.5	32.7	29.8	35.2	64.8

Furthermore, Table 4 provides the total surface free energy values for both the untreated and plasma-treated PLA scaffolds. The SFE of the untreated sample was 42.70 mN/m, which increased remarkably to 61.83 mN/m after plasma treatment. This increase in SFE enhances the surface hydrophilicity, indicating the presence of polar groups and underscoring their significant role in the plasma treatment process, which is corroborated by the XPS results.

**Table 4:** Water contact angle measurements and SFE results for the untreated and plasma-treated PLA scaffolds.

Sample	$\, \theta {\text{ (H}}_{2} {\text{O)}}$	$\theta {\text{ (CH}}_{2} {\text{I}}_{2} {)}$	$\gamma {\text{ (mN/m)}}$
Untreated PLA	92.5 ± 2°	35.5 ± 0.85°	42.70
Plasma-treated PLA	42.2 ± 1°	40.0 ± 0.45°	61.83

**Figure 7:**
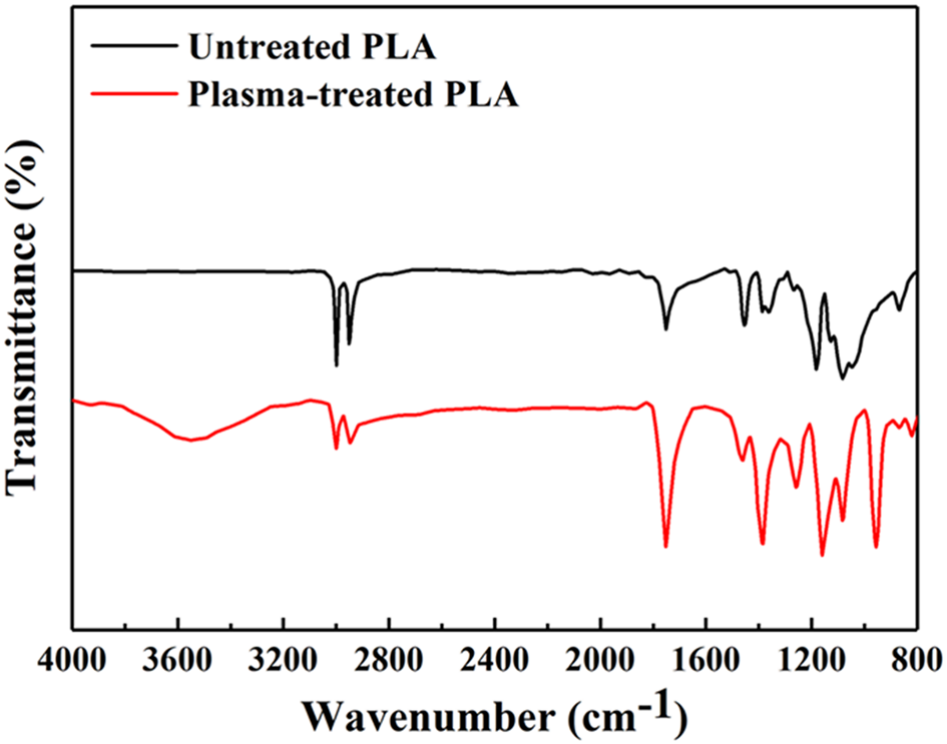
ATR-FTIR of untreated and plasma-treated PLA samples.

The augmented hydrophilicity observed in the plasma-treated samples can also be explained through the results obtained from ATR-FTIR. As presented in Fig. 7, the ATR-FTIR spectrum allows us to compare pure PLA with plasma-treated PLA samples. In the ATR-FTIR spectrum, distinct peaks are evident: the peak at 1080 cm^−1^ corresponds to –O–C=O bonding, while the peak at 1175 cm^−1^ indicates stretching –CH–O bonding. Additionally, the peak at 1265 cm^−1^ arises from C–O bonding within the ester group of PLA^39,50^. Furthermore, a well-defined peak is observed at 1745 cm^−1^, corresponding to C=O bonding. Following the plasma treatment with argon, the intensity of the oxygen-containing bonds, encompassing –O–C=O, –CH–O, C=O, and C–O, experiences a proportional increase with the number of oxygen bonds. This plasma treatment introduces ions, atoms, and excited molecules to the PLA surface, leading to surface etching and the breakage of polymer chains. Consequently, these sites interact with the ambient air, resulting in the creation of oxygen-containing compounds. In addition, upon plasma treatment, the absorption peaks between 2950 cm^−1^ and 3000 cm^−1^, associated with the –CH stretching, were observed to decrease. In this case, the –CH stretching bonds may undergo some modifications or cleavage, resulting in a decrease in their intensity in the FTIR spectrum. Moreover, the appearance of absorption peaks between 3650 and 3300 cm^−1^, at 950 cm^−1^, and an intensified peak at approximately 1375 cm^−1^ after plasma treatment, are likely attributed to OH stretching vibrations^39^. These vibrations significantly contribute in enhancing the hydrophilicity of the plasma-treated PLA samples.

### Mechanical characterization

**Figure 8:**
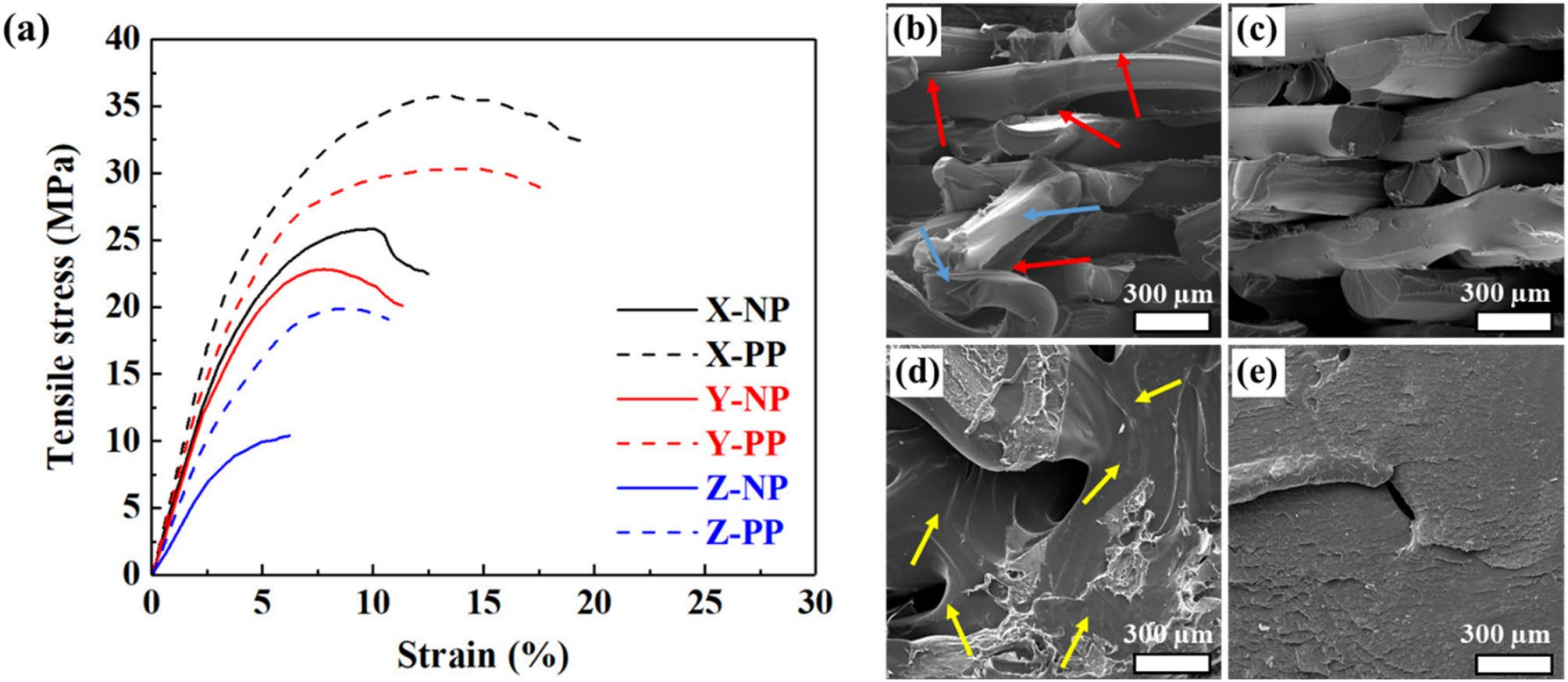
Tensile curves of the untreated and plasma-treated 3D printed PLA samples, fabricated in different orientations (**a**). FESEM fracture surfaces of the X-NP (**b**), X-PP (**c**), Z-NP (**d**), and Z-PP (**e**) samples.

One of the most important requirements of scaffolds for successful use in bone tissue engineering is possessing acceptable levels of mechanical properties. To investigate the effect of in-situ plasma treatment on mechanical properties and also anisotropy of mechanical behavior of 3D-printed PLA, tensile tests were performed on untreated and plasma-treated samples built in different orientations. The obtained tensile curves are shown in Fig. 8, while the extracted mechanical properties are summarized in Table 5. According to these results, it is clear that in all directions, samples treated with plasma show improved mechanical properties, including ultimate tensile strength (UTS) and elastic modulus, compared to untreated samples. For example, the UTS value of PLA is increased after in-situ plasma treatment by 38.6, 32.9 and 91.3% in the X, Y and Z directions, respectively. Thus, the amount of obtained improvement in mechanical properties of PLA due to application of in-situ plasma treatment is direction dependent. In addition, considerable mechanical anisotropy can be observed in both samples, where samples can be rated based on their strength level as X > Y > Z in both untreated and plasma-treated samples.

**Table 5:** Tensile test results of the untreated and plasma-treated PLA parts.

Samples	Ultimate tensile strength (MPa)	Tensile modulus (MPa)	Elongation at break (%)
X-NP	25.8 ± 3.1	569.1 ± 13.8	12.5 ± 2.4
X-PP	35.8 ± 1.5	684.6 ± 15.2	19.7 ± 1.2
Y-NP	22.8 ± 1.8	524.4 ± 17.0	11.3 ± 1.0
Y-PP	30.3 ± 2.2	619.9 ± 18.1	17.9 ± 1.7
Z-NP	10.4 ± 1	283.9 ± 20.0	6.2 ± 1.2
Z-PP	19.9 ± 1.3	447.2 ± 13.0	10.7 ± 1.4

To understand better the observed differences in mechanical response of the studied samples, fracture surfaces were investigated using FESEM, and the results are shown in Fig. 8. It is apparent from the fracture surfaces that for samples printed in the X and Y directions, failure has occurred in struts and interlayer surfaces. Struts are the thin, elongated support elements or beams that form the lattice structure within the 3D-printed sample. These struts are an integral part of the lattice pattern and play a crucial role in providing structural support and stability to the printed object. Since the majority of the force is endured by the struts, the contribution of strut breakage is more prevalent ^51^. Fracture surface of the X-NP sample demonstrates that the contact surface between the struts in adjacent layers of this sample was weak (Fig. 8b). As a result, when a force was applied, the contact interaction between struts, known as the interlayer surface, was completely obliterated in some areas, as displayed by the red arrows in Fig. 8b. These struts became more elongated following detachment, as indicated by the blue arrows in Fig. 8b. However, this is not the case with the X-PP sample shown in Fig. 8c, as the interface energy between the struts was enhanced to some extent by the use of the in-situ argon plasma treatment. As for the Z-NP and Z-PP samples, the fracture surface displayed some smooth and untouched regions (indicated by yellow arrows in Fig. 8d), suggesting that there was not enough interlayer bonding, and layers separated easily when force was applied. Interestingly, after plasma treatment, the surface became rough, indicating a better interaction surface, as shown in Fig. 8e. This resulted in acceptable mechanical bonding between layers, thereby improving tensile properties.

In contrast to the X and Y printing directions, specimens printed in the Z direction failed only from the interlayer surface^52^. The main reason for this is that in Z building direction, the applied force is perpendicular to the surface of the interlayer and is responsible for most of the mechanical response. Accordingly, increasing the interlayer area after plasma treatment in this direction resulted in greater tensile enhancement than in other directions.

**Figure 9:**
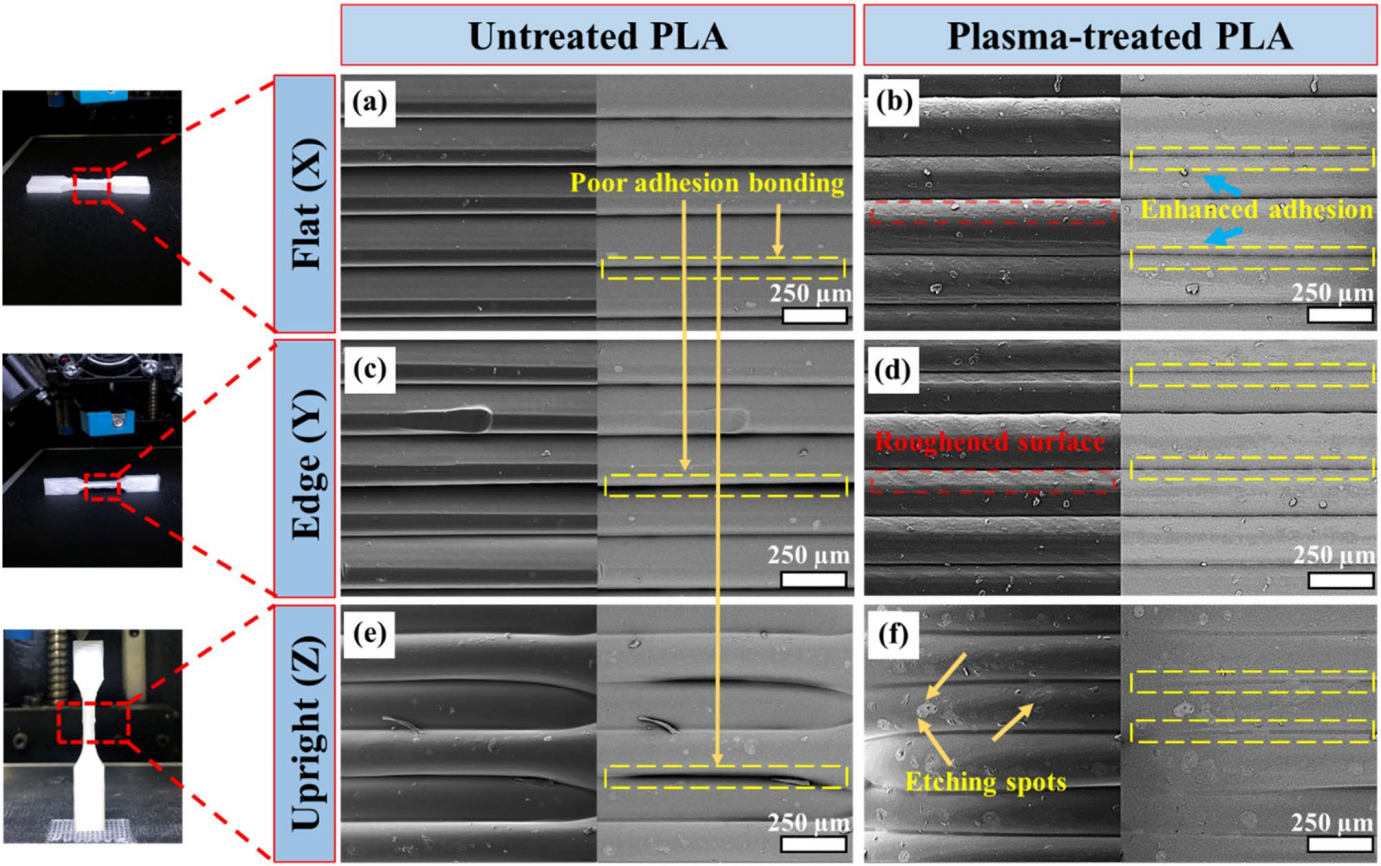
Side-view FESEM images of the untreated (**a**, **c**, **e**), and plasma-treated (**b**, **d**, **f**) 3D-printed tensile specimens.

Figure 9 depicts side views of tensile specimens for a better understanding of how plasma treatment can affect the interlayer bonding. For each sample, images were taken in both the secondary and backscatter modes (left and right, respectively). According to Fig. 9a, c and e, the untreated samples printed in the X, Y, and Z directions have a smooth surface, and also suffer from poor adhesion bonding between some of their interfaces. This weakness can be attributed to the fact that during fused deposition modeling, only localized heating of the material occurs at the nozzle. Following extrusion and prior to being laid down on top of a deposited layer, the melt begins to cool and solidify^53^. As a result of the insufficient time and thermal energy stored in the material, this process leads to partial diffusion. It is also possible that the condition of the top surface of the last deposited layer may not be conducive to bonding. It may be caused by weak boundary layers, contamination, or low surface energy^54^. Interestingly, due to surface modifications as the result of in-situ argon cold plasma treatment, the adhesion between layers is greatly improved compared with the untreated sample (Fig. 9b, d, and f). It is possible to justify the enhanced interlayer bonding of the plasma-treated samples by the following reasons. At first, plasma treatment triggers the oxidation of polymer surfaces, resulting in the formation of surface-active polar groups like carbonyl groups. As previously mentioned, the SFE value of the PLA scaffolds increased after plasma treatment due to the formation of polar groups. This enhancement in hydrophilicity is a direct result of the applied plasma treatment. The presence of these polar groups plays a significant role in transforming the surface properties, making it more hydrophilic. By virtue of improved wettability, the liquid can be spread across the surface, thereby increasing the surface area available for diffusion bonding^55^. Also, in the plasma environment, highly reactive species can abstract hydrogen atoms from the PLA polymer chains. This leads to the creation of free radicals, which are atoms or molecules with unpaired electrons. Free radicals are highly reactive and can readily participate in chemical reactions. The presence of free radicals on the PLA surface can facilitate chemical interactions with other PLA chains or with adjacent layers during the 3D-printing process.

As opposed to natural materials, polymers consist of long molecular chains that are linked end to end. These chains have only a few dangling ends at the surface for further bonding^56^. Polymers are therefore unable to adhere to each other and have low wettability. During plasma treatment, the high-energy ions and free radicals in the plasma can break the chemical bonds within the PLA polymer chains. This process is known as bond scission^57^. As a result of bond scission, some polymer chains may break into shorter segments or even individual monomer units. The formation of shorter polymer chains and monomer units can increase the number of active sites available on the surface^58^. These polymeric scission compounds promote superior interfacial flow and interdiffusion through their lower viscosities, glass transition temperatures, and lower molecular weights, resulting in reduced thermal energy (lower temperatures) required for bonding^43^. Hence, both bond scission and hydrogen abstraction result in the formation of reactive species and active sites on the PLA surface^59^. These reactive sites can promote stronger interactions and chemical bonding between PLA chains in adjacent layers during the 3D-printing process. As a consequence, the enhanced bonding between PLA layers improves the interlayer adhesion and overall mechanical properties of the 3D-printed PLA material. Secondly, plasma treatment can create a roughened surface on the struts, which can enhance mechanical interlocking and increase the surface area available for bonding^60^. The roughened surface can also increase friction between the layers, thereby improving mechanical stability and integrity. Lastly, plasma treatment can induce changes in the surface morphology of samples, such as the formation of micro/nanostructures or the removal of polluted material from layers. These changes can alter the surface properties and affect the mechanical properties of the scaffold, such as stiffness, strength, and toughness.

### In-vitro degradation

**Figure 10:**
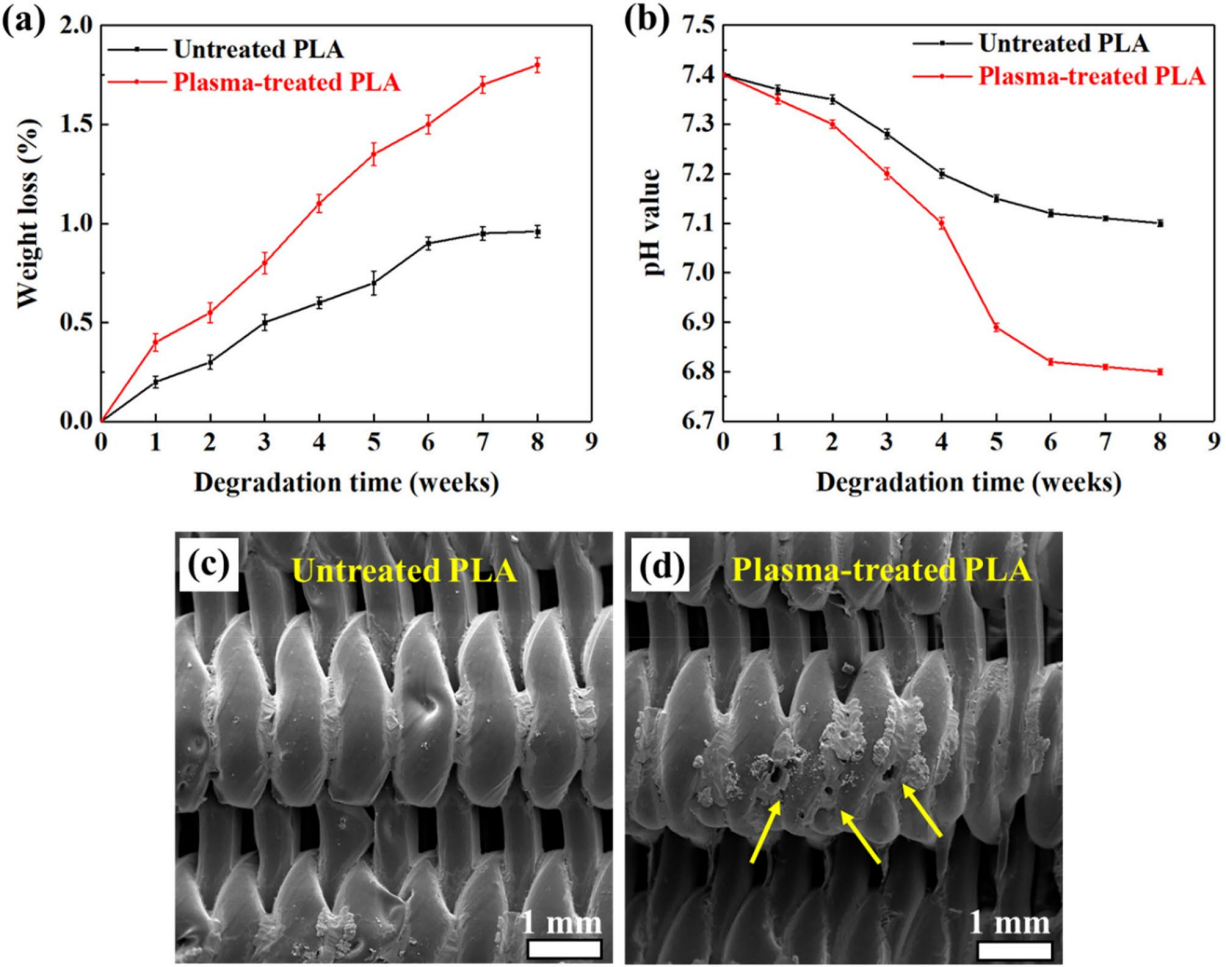
Weight loss (**a**), and pH value of the solution (**b**), recorded during 8 weeks of immersion in the BS solution, for both the untreated and plasma-treated PLA scaffolds. FESEM micrographs of the untreated (**c**), and plasma-treated (**d**) PLA scaffolds, after 8 weeks of immersion.

To achieve a successful regeneration process, tissue regeneration and scaffold degradation must be synchronized. PLA's slow degradation rate poses a serious challenge to its extensive use as a degradable substitute for bone tissue^61^. Accordingly, the effect of in-situ argon plasma treatment on the degradation behavior of 3D porous PLA scaffolds was examined in the present study. During 8 weeks of immersion in the PBS solution, scaffolds' weight loss, together with pH values of the solution, were recorded continuously every week and the obtained results are presented in Fig. 10a and b, respectively. As can be observed in Fig. 10a, weight loss of the plasma-treated scaffold is higher than the untreated sample at all immersion times, which shows higher degradation rate of the plasma-treated sample There is a significant difference between the degradation rates of the two samples, where the degradation rate of plasma-treated PLA scaffolds at eighth week is approximately 77% higher than that of untreated PLA scaffolds. As shown in Fig. 10c, surface of the untreated PLA scaffold is relatively intact after 8 weeks of immersion, while surface of the modified PLA scaffold (Fig. 10d) exhibits some pits and holes (indicated by arrows), which are indications of a more severe degradation process. The degradation of the plasma-treated PLA scaffold also resulted in a more severe decrease in the pH value of the solution (Fig. 10b), as the result of higher release rate of the lactic acid due to higher degradation rate, as discussed in the following.

**Figure 11:**
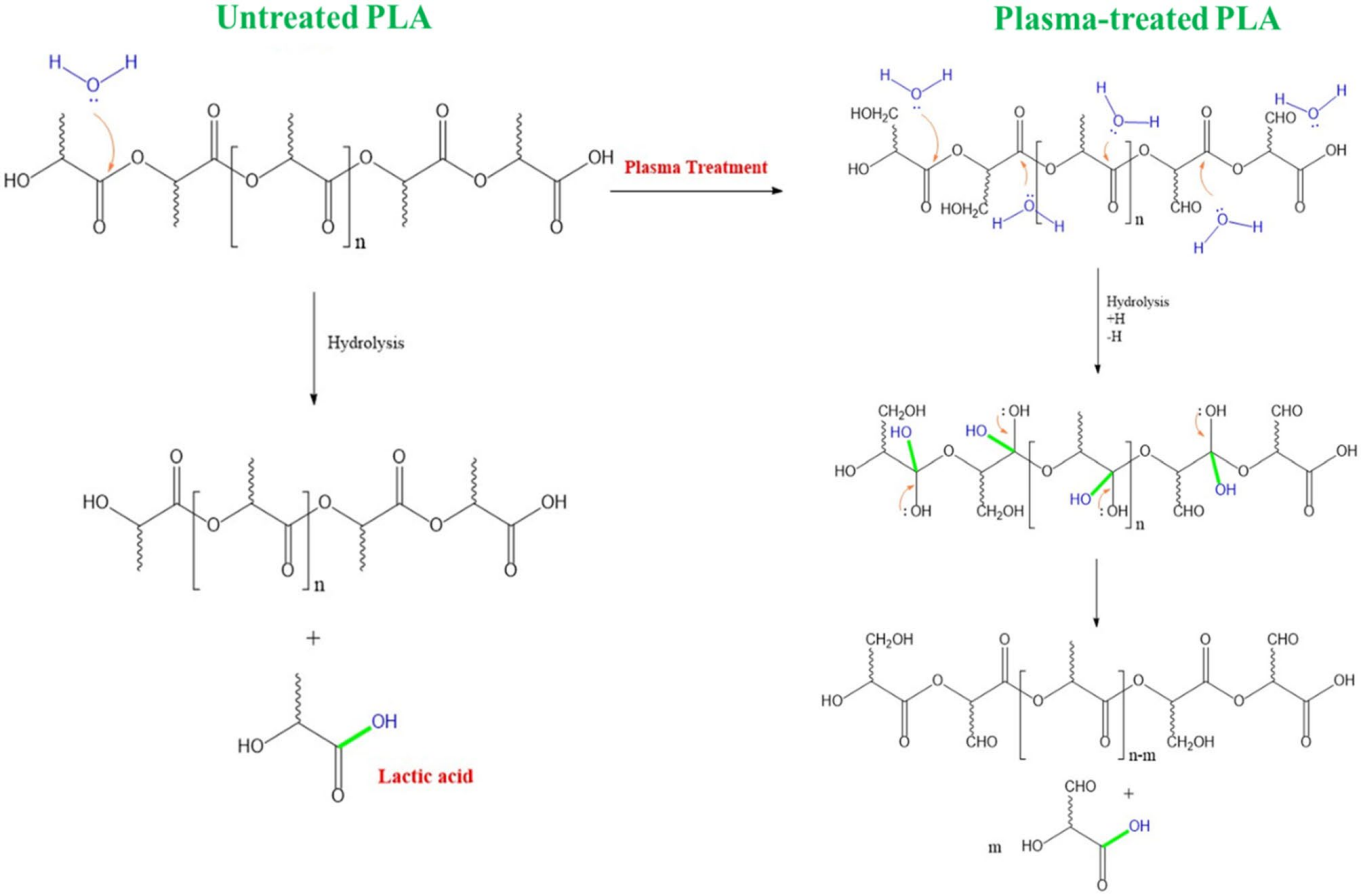
Molecular degradation mechanism of untreated and plasma-treated PLA scaffolds in the PBS solution.

The proposed mechanism for the degradation mechanism of untreated and plasma-treated PLA scaffolds is illustrated in Fig. 11. When PLA is exposed to water or a buffer solution like PBS, it undergoes hydrolysis^62^. This is the process of breaking down polymer chains into smaller units due to the presence of water molecules. PLA hydrolysis leads to the generation of lactic acid, which can decrease the pH of the surrounding environment. Plasma-treated scaffolds have enhanced wettability as a consequence of an increase in oxygen-containing bonds and also roughness. This indicates that the degradation rate of the plasma-treated PLA scaffold is superior to that of untreated one, because more water molecules would be present in this scaffold. Therefore, the pH value for plasma-treated scaffold is likely to decrease more rapidly.

### Cell viability, spreading, and proliferation

**Figure 12:**
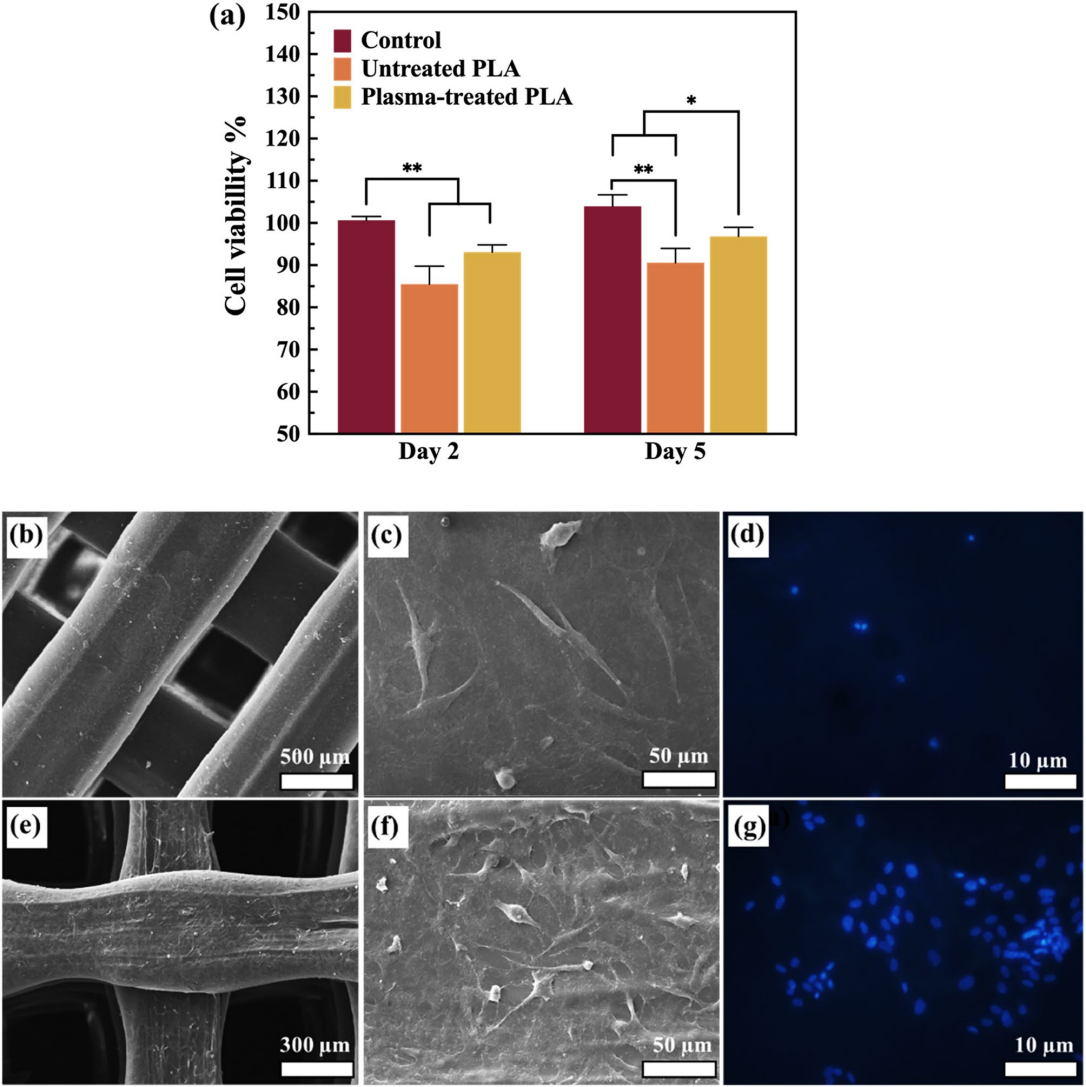
MTT assay (**a**), FESEM analysis of untreated (**b**, **c**) and plasma-treated (**e**, **f**) 3D-printed PLA scaffolds after 5 days of cell culture. DAPI staining of untreated (**d**) and plasma-treated (**g**) PLA scaffolds. (*p < 0.05, **p < 0.01).

An MTT assay was performed to evaluate the viability of hADSCs cultured on both untreated and plasma-treated PLA scaffolds after two and five days. Based on the analysis of cell viability presented in Fig. 12a, the plasma-treated PLA scaffolds exhibit a higher level of cell viability than the untreated ones, for two and five days. While the difference in the cell viability percentage between the untreated PLA and treated one was about 3% in two days, this amount doubled after five days and the cell viability of the modified PLA scaffold reached 96.13 ± 3.44%. These results are consistent with recent literature which investigated the effect of oxygen and nitrogen plasma treatment on PCL/HA/MgO scaffolds^38^. In this regard, it has been reported that cell growth and proliferation were enhanced without non-inflammatory effects^63, 64^. Therefore, 3D-printed PLA scaffolds treated with in-situ argon plasma may exhibit greater bioactivity than pure PLA.

FESEM analysis was employed to examine the effect of plasma treatment on hADSCs' adhesion morphology. As shown in Fig. 12b and c, cells on the untreated PLA surface displayed a flat and fusiform spindle shape. In contrast, plasma-treated PLA scaffolds showed irregularly shaped hADSCs with dispersed cytoplasm (Fig. 12e and f). The hADSCs grown on untreated PLA scaffolds exhibited fewer flattened spindle-like patterns, whereas the hADSCs on plasma-exposed scaffolds developed more polygonal shapes, particularly at higher magnification. Moreover, after five days of culture, the number of hADSCs on plasma-treated PLA scaffolds increased markedly compared to those on untreated scaffolds. DAPI is widely used as a fluorescent stain to identify DNA in both live and fixed cells, as it can bind to specific regions in DNA^65^. After seeding the cells on scaffolds, DAPI staining was used to evaluate their morphological properties, as shown in Fig. 12d and g. From this figure, it is evident that plasma-treated PLA scaffolds show a higher rate of cell spreading.

## Conclusion

This study aimed to enhance the physio-mechanical and biological features of 3D-printed PLA scaffolds using in-situ argon plasma treatment. The AFM results revealed a significant increase in the root mean square roughness value after plasma modification, rising from 1.5 nm to 70 nm. Additionally, XPS analysis demonstrated an increase in single and double carbon–oxygen bonds, specifically C–O and O–C=O, on the surface of the plasma-treated sample, indicating creation of new chemical bonds. This resulted in a remarkable increase in hydrophilicity, with the contact angle decreasing from 92° to 42.5° after plasma treatment. Furthermore, tensile properties of the plasma-treated PLA specimens were significantly improved in all printing directions, particularly in the Z direction. The ultimate tensile strength and elastic modulus showed substantial enhancements, escalating from 10.4 MPa and 283.9 MPa to 19.9 MPa and 447.2 MPa, respectively. In the in-vitro degradation study conducted in PBS, the plasma-treated PLA scaffold exhibited 77% higher degradation within 8 weeks compared to the untreated scaffold. Moreover, the modified scaffold exhibited improved viability, attachment, and proliferation of human adipose-derived stem cells. In conclusion, the obtained results highlight that in-situ argon cold plasma treatment is a cost-effective and facile method for modifying and improving different properties of PLA scaffolds to suit specific applications, particularly in the field of bone tissue engineering.

## Data Availability

The datasets generated during and/or analyzed during the current study are available from the corresponding author on reasonable request.
